# Patients as teachers: a qualitative study of spiritual care delivery experiences of senior healthcare providers in Taiwan

**DOI:** 10.1186/s12909-026-08852-1

**Published:** 2026-02-24

**Authors:** Hsiao-Fen Lo, Jaw-Shiun Tsai, Chen-Yin Tung, Chia-Chen Chang

**Affiliations:** 1 Department of Health Promotion and Health Education, College of Education, National Taiwan Normal University, No. 162, Section 1, Heping E. Rd., Taipei, 106 Taiwan, ROC; 2 Department of Family Medicine, College of Medicine, National Taiwan University, No. 7 Chung-Shan South Road, Taipei, 100 Taiwan, ROC; 3 Department of Family Medicine, National Taiwan University Hospital, No. 7 Chung-Shan South Road, Taipei, 100 Taiwan, ROC; 4 Department of Family Medicine, Jin-Shan Branch, National Taiwan University Hospital, New Taipei City, Taiwan, ROC

**Keywords:** patient-as-teacher, spiritual care, cultural sensitivity, healthcare professional, reflective practice, professional growth

## Abstract

**Background:**

Spiritual care is a core component of holistic health care that addresses patients’ existential and emotional needs. However, in Taiwan, spiritual care delivery remains inconsistent due to cultural, educational, and institutional barriers. This study explored the spiritual care delivery experiences and reflections of healthcare professionals in Taiwan.

**Methods:**

Focus group interviews were conducted with 15 physicians and nurses from healthcare institutions across northern Taiwan. Interview data were subjected to thematic analysis to identify key patterns and themes.

**Results:**

Four major themes emerged: (1) subtle and culturally mediated manifestations of spiritual distress, requiring heightened clinical sensitivity; (2) trust-building as a foundation for spiritual dialogue; (3) facilitating meaning reconstruction through spiritual care delivery; and (4) reflection and integration into professional learning and personal growth. Participants emphasized the need for culturally sensitive education, experiential learning methods, and institutional support to strengthen spiritual care practices.

**Conclusions:**

Spiritual care is a reciprocal and transformative process that benefits both patients and caregivers. Healthcare institutions and training programs should integrate cultural sensitivity, reflective practice, and experiential learning into education and policy to help professionals effectively address patients’ spiritual needs.

**Supplementary Information:**

The online version contains supplementary material available at 10.1186/s12909-026-08852-1.

## Background

Spiritual well-being has emerged as a key component of global health frameworks, particularly in serious illness and end-of-life care [1, 2]. Spirituality strengthens emotional resilience and fosters positive psychological states, such as gratitude and a sense of purpose [3, 4]. Spiritual support improves quality of life, promotes emotional adjustment, and facilitates decision-making among patients and their families [5–9]. Researchers have proposed multiple conceptual definitions of spiritual health, but a universal definition remains to be established [10–16]. Spirituality can be broadly understood as the dimension of human existence through which “individuals seek and express meaning and purpose, and the way they experience their connectedness to the moment, to self, to others, to nature, and to the significant or sacred” [16]. In the present study, spirituality was defined as the process through which individuals seek meaning, connection, and inner peace amid life’s challenges and physical, emotional, and social concerns.

Cultural context complicates the concept of spiritual health. In Chinese-speaking countries such as Taiwan, spiritual distress is often subtly, vaguely, or ambiguously expressed [17]. In some traditional cultures, spirituality-related topics—particularly death—are often considered taboo or inauspicious; in such cultures, patients and their families remain silent or avoid discussing existential struggles [18–22]. Similarly, clinicians may avoid raising spiritual concerns for fear of intrusion or offense [14, 17, 20, 23]. Therefore, spiritual distress is difficult to detect without specific training. In many Asian societies, spiritual needs are interpreted through the lens of family responsibility, ancestral beliefs, or moral obligation; this approach differs from the emphasis on individual autonomy in Western societies. These cultural dynamics suggest that spiritual care delivery cannot be understood without attention to locally embedded meanings.

Although healthcare providers generally acknowledge the importance of spirituality, they often have low competence and confidence in addressing spiritual needs [6, 24, 25]. Integration of spiritual care into clinical practice remains inconsistent due to barriers such as unclear definitions, lack of formal training, insufficient institutional support, and systemic factors (e.g., time pressure, workload, and hospital’s ethical climate) [6, 26–28]. The absence of structured procedures and scarcity of opportunities for reflection further reduce clinical professionals’ readiness to respond [25, 29].

Although scholars have described knowledge, attitudes, and behaviors related to spiritual care [30–32], few have systematically analyzed its professional and personal effects on healthcare providers. Several studies have suggested that spiritual care fosters resilience, empathy, and professional identity among clinicians [30–33]. However, how clinicians in specific cultural contexts—for example, Taiwan—recognize, interpret, and respond to patients’ spiritual concerns remains underexplored.

The present study investigated how Taiwanese healthcare professionals with significant clinical experience (≥ 10 years) address patients’ spiritual needs, interpret professional-patient interactions, and reflect on their practice. By focusing on the reciprocal nature of patient–provider interactions, this study revealed the role of cultural context in shaping spiritual care delivery and its implications for professional growth and education.

## Methods

### Study design

This qualitative study adopted the thematic analysis approach proposed by Braun and Clarke [34] to determine how Taiwanese healthcare professionals perceive, practice, and reflect on spiritual care. Thematic analysis was selected because of its flexibility and suitability for identifying, analyzing, and interpreting patterns in qualitative data, particularly those pertaining to complex and culturally embedded practices such as spiritual care. This approach allows for a comprehensive understanding of healthcare providers’ experiences without the need for formal theoretical model construction.

### Study cohort

Physicians and nurses with at least 10 years of significant clinical experience in providing clinical care were recruited from hospitals and clinics across four major cities in northern Taiwan: Taipei, New Taipei, Keelung, and Taoyuan. Two profession-specific focus groups were conducted: one with physicians (*n* = 9) and one with nurses (*n* = 6). On the basis of a review of the literature review, the researchers adopted the following key concept: spiritual health is the right of every person. Spiritual care is essential and relevant across specialties, and it should not be restricted to mental or terminal illnesses. Thus, the study sample included men and women from diverse clinical specialties and institutional backgrounds, capturing a broad range of perspectives and experiences. Demographic characteristics of the study cohort are summarized in Table 1.

**Table 1:** Demographic characteristics of the study cohort

Items	Physician (*P*)		Nurse (*N*)		Total	
	(*n* = 9)		(*n* = 6)		(*N* = 15)	
	Mean	Range	Mean	Range	Mean	Range
Gender						
Male	4	(-)	0	(-)	4	(-)
Female	5	(-)	6	(-)	11	(-)
Age (years old)	47.5	35–58	36.4	31–47	43.06	31–58
Work experience (years)	20.5	10–30	15	10–27	18.3	10–30
Specialty*	FM/PC: 4	OBGYN: 1	PC:2	OBGYN: 1	FM/PC: 6	OBGYN: 2
	M: 1	S: 1	M: 1	S:1	M: 2	S: 2
	ER:1	TCM:1	ICU:1		ER:1	TCM:1
					ICU:1	

*: *P* Physician, *N* Nurse, *FM* Family medicine, *PC* Palliative care unit, *OBGYN* Obstetrics and gynecology, *M* Internal medicine, *S* Surgery, *ER* Emergency room, *TCM* Traditional Chinese medicine, *ICU* Intensive care unit

### Data collection

Relevant data were collected in August 2024 through two semi-structured focus group interviews—one with nine physicians and the other with six nurses. Each focus group session lasted approximately 120 min and was guided by a set of open-ended questions (Appendix 1) designed to explore participants’ experiences with and reflections on spiritual care. All interviews were audio-recorded with participants’ consent and transcribed verbatim. Interview data were anonymized and cross-verified for accuracy to ensure integrity. Follow-up phone calls were initiated when the transcript did not match the notes taken by the interviewer. These steps ensured that the data accurately captured participants’ narratives and reflections, providing a robust foundation for thematic analysis.

### Data analysis

Thematic analysis was performed using the six-phase framework outlined by Braun and Clarke [34]. This framework includes the following steps: [1] familiarization with the data through repeated transcript readings [2], inductive generation of initial codes to capture major features of the data [3], grouping of codes into broader categories to identify themes [4], review of themes to ensure coherence and consistency with the data [5], conceptualization and naming of themes to articulate their essence, and [6] integration of themes into a meaningful narrative to prepare the final report.

The analysis followed a noniterative approach, in which a fixed interview guide and prompts were used to ensure consistency and data comparability. During analysis, a constant comparative method was used to identify patterns and themes within and across transcripts. Analytical insights were documented through memo-writing. Data saturation was reached when no new themes emerged.

### Cultural context and reflexivity

To ensure cultural sensitivity and maintain rigor and transparency, the research team adopted a reflexive approach that considered Taiwan’s sociocultural context, where spirituality is often closely linked to traditional values and beliefs. The primary interviewers were registered nurses and researchers with extensive experience in palliative care and spiritual research and deep insight into Taiwan’s clinical context, which facilitated meaningful dialogue with participants. The interviewers maintained neutrality by adhering to the predesigned guide and script, avoiding evaluative comments, and engaging in reflexivity to minimize potential bias.

The interview guide was developed by the research team to elicit rich and authentic responses. To ensure clarity, neutrality, and cultural relevance, two clinical experts and two senior qualitative researchers reviewed and refined the guide’s wording, structure, and flow.

During each focus group interview, the interviewer used predetermined prompts to encourage dialogue and consciously minimize personal influence over participants’ views. This approach fostered a comfortable and open environment that supported honest expression without fear of judgment.

To further support reflexivity, the research team held regular debriefings during data collection and analysis to reflect on the potential influence of the interviewer’s background. These reflections allowed the research team to identify and reduce bias, ensuring that the findings accurately represented participants’ experiences.

### Rigor and ethics

#### Rigor

Multiple strategies were implemented to ensure analytical rigor. To increase credibility, physicians’ and nurses’ perspectives were compared to triangulate interview data, supplemented by peer debriefing and member verification with select participants. To ensure dependability, a transparent audit trail comprising coded transcripts and analytic memos was maintained to document analytic decisions. Rich contextual descriptions of participants’ demographic characteristics, clinical settings, and cultural backgrounds supported transferability, enabling readers to assess the applicability of the findings to other contexts. Confirmability was strengthened through reflexive memo-writing and regular team discussions to minimize researcher bias and increase analytical transparency.

#### Ethics

The study protocol was approved by the Institutional Review Board of National Taiwan Normal University (approval number: 202312HS022). All participants provided written informed consent. They were assured that participation was voluntary and that confidentiality of their data would be maintained.

## Results

Four major themes and their subthemes were identified. The subthemes are explicitly listed in the narrative below; Additional file 1 (Table S2) is provided only as a summary for ease of reference. These themes capture how providers perceive, practice, and integrate spiritual care into clinical care in Taiwan. The four major themes were as follows: (1) subtle and culturally mediated manifestations of spiritual distress (2), trust-building as a foundation for spiritual dialogue (3), facilitating meaning reconstruction through spiritual care delivery, and (4) reflection and integration into professional learning and personal growth.

### Subtle and culturally mediated manifestations of spiritual distress

This theme comprised four subthemes: (1) uncontrolled symptoms with unknown cause (2), disorganized expressions revealing inner chaos (3), illness interpreted as punishment or burden, and (4) culturally mediated expressions of spiritual distress. Spiritual distress frequently manifested in forms that were difficult to detect using standard clinical tools. Participants reported that patients’ words, behaviors, and emotions often concealed deep existential concerns, requiring caregivers to draw on observation, clinical experience, and intuitive judgment to identify underlying spiritual needs.

#### Uncontrolled symptoms with unknown cause

Physicians observed that some patients presented with unexplained symptoms suggestive of nonphysical distress. One physician described such a case: “Despite pharmacological and counseling interventions, nothing really worked…it felt like something beyond the physical was happening” (P9, p.17). Another participant reflected on how cultural beliefs surrounding health and illness often shape patients’ expressions of distress: “Patients sometimes believe their symptoms are a sign of punishment or imbalance in their family’s spiritual harmony” (P3, p.12). These accounts highlight the complex interplay between physical symptoms and spiritual or cultural dimensions of distress and point to the need for holistic care approaches.

#### Disorganized expressions as a sign of inner chaos

Patients often conveyed existential concerns through fragmented or disorganized expressions. For example, providers described patients’ sudden, seemingly irrelevant questions and fragmented speech: “Why are you here? You should be alive.” (N2, p.59); “Why are you so tired from a tiring job while I am so tired from lying down?” (P6, p.6). Describing a patient, one nurse said, “He couldn’t get to the point—deep down, he just didn’t know how to face his situation” (N3, p.61). These observations suggest that fragmented or disorganized expressions may reflect deeper existential struggles and that some patients may require professional support to process their distress.

#### Interpretation of illness as punishment or burden

Some patients framed their illness in moral or relational terms involving guilt or punishment. A nurse described hearing a patient say, “I have to accept this punishment from God because I’m not a good person” (N6, p.69). Other patients avoided treatment to reduce the burden on their families: “If I continue to accept treatments, I will drag my family down” (P7, p.22). A nurse noted that this perspective is often rooted in traditional values: “Many patients see their illness as a burden on their family members that alters their original lifestyle” (N5, p.78). These accounts underscore the influence of cultural and moral beliefs on patients’ interpretations of illness and indicate that additional support may be needed to address associated distress.

#### Culturally mediated expressions of spiritual distress

Cultural norms and practices deeply influenced how spiritual distress was expressed, which could complicate recognition by healthcare providers. For instance, one nurse shared, “In our culture, as some participants observed, some patients—especially men—are often reluctant to express their fears or pains directly. Instead, they might talk about unrelated topics” (N5, p.75). A physician observed, “Some patients rely on religious rituals or traditional healers to address their concerns, but they may hesitate to share this information with us” (P4, p.32). Another provider noted that family members could also inhibit expression: “Sometimes, barriers stem from the family. When patients mention death-related topics, many family members react by saying, ‘Don’t think too much, you’ll get well soon,’ interrupting the patients to express themselves.” (P9, p.34). These observations are further discussed in relation to Taiwanese/Chinese cultural contexts and existing literature.

### Trust-building as a foundation for spiritual dialogue

This theme comprised two subthemes: (1) expressing needs in casual conversation and (2) presence and familiarity as essential elements. Participants emphasized that spiritual care develops gradually within a secure and trusting environment. Once caregivers identify distress, openness, presence, and attentiveness can build trust and deepen dialogue with patients.

#### Expressing needs in casual conversations

Existential concerns often surfaced in informal exchanges. For example, a nurse shared that during assisted bathing, a patient asked, “After I die, who will remember me? Will I just disappear?” (N2, p.61). Another nurse described taking a patient out to sunbathe, who suddenly asked, “After a person dies, is it really true that nothing is left behind?” (N4, p.62). Such moments can serve as entry points for spiritual dialogue, highlighting the need for providers to attend to patients’ concerns beyond formal assessments.

#### Essential roles of presence and familiarity

Continuity of care and familiarity helped to build trust. A nurse shared the following patient feedback: “It’s hard to know who I can talk to. Your staff shifts are constantly changing, and I don’t even recognize anyone” (N3, p.62). One physician described, “At first, she said nothing…After I visited every day, she finally asked, ‘Where do you think people go when they die?’” (P5, p.41). These accounts highlight the role of consistent presence in establishing trust and enabling spiritual dialogue.

### Facilitating meaning reconstruction through spiritual care delivery

This theme comprised four subthemes: (1) caregivers’ responses shape the depth of the dialogue (2), life review for integration and value reconstruction (3), symbolic actions and rituals, and (4) reconnection with others. Once patients initiated spiritual dialogue, caregivers supported meaning reconstruction through attentive listening, guided reflection, symbolic actions, and reconciliation.

#### Caregiver responses shape the depth of dialogue

Attentive listening and respectful attitudes were foundational to deepening patient engagement. One physician described listening attentively to a patient: “I didn’t give advice—just listened to him talk about his father. Later, he said, ‘I think I can forgive him now’” (P5, p.43). Similarly, a nurse encouraged a patient to keep a diary; the patient later said, “I still have something worth recording” (N3, p.61). Another nurse encouraged a patient who had been a teacher for 45 years to share her feelings after being visited by former students. The patient said, “As a teacher, at this moment, I feel grateful, my life is valuable” (N1, p.81). These examples illustrate how communication skills and a respectful stance can create a safe space for patients to explore meaning, connection, and value, thereby supporting emotional healing and personal growth.

#### Life review for integration and value reconstruction

Guided reflection on past experiences helped patients recognize their worth and find meaning. A physician shared the following patient comment: “Some of my students visited me after I got sick. Now I realize that my companionship in those days was meaningful” (P9, p.38). A nurse described a patient’s self-affirmation of “I’m a useful person” after reflecting on a lifetime of caregiving (N5, p.72). These accounts suggest that life review can help patients integrate prior experiences, reconstruct their sense of value, and sustain meaning despite illness.

#### Symbolic actions and rituals

Symbolic acts allowed patients to express emotions and affirm identities. One nurse’s patient wrote a letter to a deceased loved one, saying, “I wanted her to know I’m doing okay,” (N4, p.62). Another physician had patients plant trees as a legacy for their children; one patient remarked, “This tree symbolizes me accompanying my children as they grow” (P7, p.35). These findings highlight how symbolic actions and rituals can support emotional processing, identity affirmation, and legacy building.

#### Reconnection with others

Patients often experienced transformation through reconciliation with loved ones. One physician’s patient expressed, “I want to apologize to my son for being too strict” (P9, p.19). A nurse’s patient, initially resistant to visits, later told his wife, “I want you to know I’m trying very hard to live” (N1, p.84). These examples underscore the role of reconnection in fostering emotional healing and strengthening relationships during illness.

### Reflection and integration into professional learning and personal growth

This theme comprised three subthemes: (1) patients as teachers for professional and personal growth (2), redefining professional roles and mission, and (3) addressing challenges and educational needs. Spiritual care delivery not only supported patients but also shaped caregivers’ professional learning. Participants described patients as teachers who inspired reflection, transformed clinicians’ understanding of care, and reinforced their professional mission.

#### Patients as teachers for professional and personal growth

Clinical encounters often served as profound learning experiences, particularly when formal education provided limited preparation for spiritual care. Most participants reported gradually developing skills and confidence through patient interactions, learning from patients’ reactions and feedback. For instance, one physician reflected on a painful experience: “I was shocked when I heard the patient tell my colleague that she hated me…I later realized she felt abandoned because she didn’t see me until I left. That’s when I learned how important presence is, even just saying hello” (P6, p.42). Another physician shared that a patient “taught me what it means to face life bravely. I was not just his doctor—I was his student” (P6, p.38). These accounts highlight the reciprocal nature of spiritual care, in which patients act as co-educators shaping clinicians’ reflective practices.

#### Redefining professional roles and missions

Spiritual care prompted clinicians to broaden their roles and offer holistic presence beyond disease treatment. A physician noted, “I realized that what truly matters is presence and listening” (P5, p.7). Similarly, a nurse shared, “Medical care is about not only treatment but also connection and companionship” (N3, p.70). Engagement in spiritual care encouraged clinicians to redefine their professional roles, shifting from a sole focus on disease treatment to a more holistic approach emphasizing presence, connection, and compassionate companionship.

#### Addressing challenges and educational needs

Participants identified systemic and cultural challenges in spiritual care delivery, including low confidence, limited experience, and concerns about cultural sensitivity. A physician remarked, “Daily clinical schedules are demanding, involving practice, teaching, and research. Most of the time, spiritual care isn’t my priority” (P3, p.11). A nurse shared, “Sometimes, when patients talk about their beliefs, I don’t know how to respond. I worry my response might offend them” (N2, p.73). To address these challenges, participants emphasized the need for structured education that integrates cultural awareness. A physician suggested, “Practicing through case simulations or role-play during on-the-job training or even earlier in school would give us more confidence” (P4, p.39). These examples illustrate the need to address systemic and cultural barriers through structured education and training.

## Discussion

This study explored spiritual care–related clinical experiences and reflections of healthcare providers in Taiwan. Focus group interviews led to the identification of four key themes related to spiritual care delivery: recognition of spiritual distress, cultural challenges, bidirectional transformation, and educational implications.

### Recognition of spiritual distress

Participants’ experiences highlighted the diversity and subtlety of patients’ spiritual needs. In clinical practice, spiritual distress often manifests subtly or nonverbally. When clinical sensitivity is insufficient, these cues are often overlooked. Prior studies have reported that healthcare providers often receive limited or no training in recognizing and addressing spiritual distress [7, 31, 35].

Participants in the present study emphasized the need to develop observational and reflective skills to detect nuanced expressions of distress.

### Cultural challenges in spiritual care

Within Taiwanese society, death and spirituality are often considered taboo or private topics, which can prevent patients and their families from openly expressing spiritual concerns.

Beyond taboo-related avoidance, our findings indicate that spiritual distress may be communicated indirectly and shaped by gender and family dynamics. Providers noted that some patients—particularly men—shifted to unrelated topics rather than articulating fear or suffering, and that patients who relied on religious rituals or traditional healers sometimes hesitated to disclose this to clinicians. Family members could further constrain disclosure by interrupting death-related talk. These patterns align with Taiwanese literature on gender-related communication needs and family-centered barriers in serious illness and end-of-life contexts [36–38], underscoring the need for culturally competent, permission-giving communication and sensitive negotiation of family presence. At the same time, the lack of culturally grounded training increases provider uncertainty regarding how to approach these concerns, a challenge identified in numerous studies [39, 40]. Similar barriers have also been documented internationally, where institutional constraints and limited time for spiritual conversations impose additional challenges [27–29, 41].

These studies highlight the need for culturally sensitive approaches that incorporate cultural humility and can adapt to local norms and values.

### Bidirectional transformation in spiritual care

Participants’ experiences with spiritual care delivery were strongly shaped by their reciprocal interactions with patients. These encounters not only support patients’ spiritual well-being but also prompt caregivers’ self-reflection, personal growth, and professional development. Participants described learning from patients how to create a safe and trusting environment that fosters openness and deepens spiritual connection.

The theme of *patients as teachers* is a key insight of this study, illustrating how addressing patients’ spiritual needs prompted participants to reassess their own values, strengthened their resilience, and reaffirmed their sense of professional duty. This reciprocal dynamic reinforces evidence emphasizing the bidirectional nature of spiritual care [32, 42–44], wherein both patients and healthcare providers engage in mutual learning and transformation.

The reciprocal nature of spiritual care positions it not only as a clinical practice but also as a relational and humanistic encounter that nurtures empathy and broadens clinicians’ understanding of their roles. The findings of this study reinforce prior evidence and highlight the need for further exploration of this reciprocal dynamic as a framework for understanding professional growth in relation to spiritual care practices.

### Educational implications and future curriculum development

Participants reported the general absence of spiritual care education in their previous academic and clinical training. They identified specific educational needs and emphasized the value of experiential learning approaches. The development of spiritual care competencies is a key priority in health professional education, particularly because spiritual health concerns extend beyond palliative or mental health contexts to encompass the holistic well-being of all patients.

Participants’ experiences indicated that trust and rapport in spiritual care are cultivated gradually through continuity, relational safety, and reflective engagement. Patients who feel secure are more willing to share their inner beliefs and fears. Accordingly, medical and nursing education and on-the-job training for junior staff should cultivate core relational skills, such as sustained presence, reflective listening, and guided life review, to strengthen clinicians’ ability to build trust and address patients’ spiritual needs effectively.

Curricula must also integrate cultural sensitivity and patient narratives. Training programs should equip healthcare professionals to navigate the spiritual and cultural diversity they will encounter in clinical practice, fostering culturally responsive communication and empathy. Educational emphasis on cultural humility enables clinicians to deliver spiritual care that is both respectful and meaningful in diverse patient contexts.

The present study highlights the reciprocal dimension of spiritual care, in which patients act as co-educators who inform clinicians’ reflective and ethical awareness. This reciprocity indicates that spiritual encounters are inherently mutual learning experiences rather than unidirectional acts of care. To translate this insight into education, experiential learning strategies, such as role-play, guided reflection, and case-based simulation, should be embedded in training programs. These methods provide dynamic opportunities for learners to internalize the reciprocal processes of giving and receiving in spiritual care, deepening relational awareness and professional maturity.

Spiritual care education should be systematically reconceptualized as both a formative and transformative learning process rather than a discrete skill set. Educational programs can engage learners in continual self-reflection, meaning-making, and demonstrations of cultural humility to ensure healthcare professionals are not only clinically competent but also spiritually attuned and capable of providing holistic, person-centered care.

Future studies should investigate how reciprocal learning processes involving patients as co-educators can be systematically embedded in pedagogical frameworks. Longitudinal and cross-cultural studies are required to elucidate how such educational innovations influence professional identity formation, resilience, empathy, and the overall quality of holistic care.

### Implications for curriculum development

This study highlights the need for structured, longitudinal integration of spiritual care education into healthcare curricula. Future curriculum design should extend beyond theoretical instruction to incorporate experiential, reflective, and interprofessional dimensions to cultivate both competence and compassion. Spiritual care competencies can be embedded within communication, clinical ethics, and palliative care modules to further ensure relevance and continuity throughout medical and nursing training.

### Limitations

#### This study has several limitations

First, participants were recruited from healthcare institutions in northern Taiwan, which may limit the transferability of the findings to other regions or healthcare systems.

Second, participants were required to have at least 10 years of clinical experience to ensure sufficient practical exposure to spiritual care delivery. However, this criterion meant the perspectives of junior staff were excluded. Consequently, the findings mainly reflect the perspectives of mid- to senior-level professionals, potentially overlooking the unique experiences and challenges of less experienced staff. Future research should include junior healthcare providers to capture spiritual care practices across career stages.

Third, the small sample size (*n* = 15) warrants caution regarding the generalizability of the results to the broader population of healthcare providers in Taiwan.

Finally, although focus group interviews facilitated interaction and reflection, group dynamics may have influenced participants’ openness in sharing and introduced social desirability bias.

## Conclusions

This study highlights the reciprocal nature of spiritual care, wherein patients serve as co-educators who inspire clinicians’ reflection, growth, and sense of purpose. By attending to patients’ spiritual needs, healthcare providers also cultivate self-awareness and professional development. This process positions spiritual care as a humanistic practice grounded in cultural and relational contexts. Future educational and policy initiatives should foster context-specific competencies integrating cultural sensitivity, experiential learning, and reflective practice. Further research involving patients, family members, and trainees is required to deepen the understanding of the broader effects of spiritual care.

## Supplementary Information

Supplementary Material 1.

Supplementary Material 2.

Supplementary Material 3.

## Data Availability

The data sets generated and analyzed in this study are available from the corresponding author on reasonable request.
