# Supplementary material for: Patients as teachers: a qualitative study of spiritual care delivery experiences of senior healthcare providers in Taiwan
Source: BMC Med Educ. 2026 Feb 24;26:523. doi: 10.1186/s12909-026-08852-1 (PMC13037104; doi:10.1186/s12909-026-08852-1)
Supplement: Supplementary file 1 — Supplementary Material 1. [file 12909_2026_8852_MOESM1_ESM.pdf]

Appendix 1：Interview guide

【Focus Group Interview Guide – Healthcare Professionals】

Purpose:

This study aims to understand the perspectives and practical experiences of healthcare professionals in the field of spiritual care, providing insights for the development of spiritual care competence and educational courses.

Interview Questions:

1. **Concept of Spiritual Health and Spiritual Distress**

(1) What does spiritual health mean to you?

(2) Do you think spiritual health is important? Why or why not?

(3) In your opinion, what does spiritual health mean for patients? Is it essential to address this issue? Why?

(4) Is there a connection between healthcare professionals' spiritual health and patients' spiritual health? Why or why not?

(5) Based on your experience, what are the potential consequences for patients with spiritual distress? How might it affect them?

2. **Competencies of Spiritual Care**

(1) Can you share your experiences caring for patients with spiritual distress? (e.g., How did you identify the issue? How did you build relationships with

the patient? What strategies did you use? What changes or feedback did the patient show? What are your personal and professional reflections on your caring experiences? Please describe the process.)

(2) What challenges do you think healthcare professionals face when caring for patients with spiritual distress? (e.g., If it was a challenge, what did you do? How did you feel? Please describe the process.)

(3) What key competencies do healthcare professionals need in providing spiritual care?

(4) What are the educational needs for improving spiritual care competencies?

Note:

✓Please include basic demographic information (e.g., age, gender, background) when sharing case experiences.

✓Each participant is encouraged to share their thoughts within 2–3 minutes per question.
