# Supplementary material for: Patients as teachers: a qualitative study of spiritual care delivery experiences of senior healthcare providers in Taiwan
Source: BMC Med Educ. 2026 Feb 24;26:523. doi: 10.1186/s12909-026-08852-1 (PMC13037104; doi:10.1186/s12909-026-08852-1)
Supplement: Supplementary file 2 — Supplementary Material 2. [file 12909_2026_8852_MOESM2_ESM.pdf]

Wallace Academic Editing

English Editing Certificate

This certifies that the paper **Patients as Teachers: A Qualitative Study of Spiritual Care Delivery Experiences of Senior Health-Care Providers in Taiwan** has been edited by Juliet Paulson on February 6, 2026 and is considered to be improved in grammar, punctuation, spelling, verb usage, sentence structure, conciseness, general readability, writing style, and native English usage to the best of the editor's ability.

Juliet Paulson

Best regards,
Wallace Academic Editing

Phone No.: +886-2-2555-5830
Website: http://www.editing.tw
Email: editing@editing.tw
Address: 6F.-8, No. 287, Chang'an W. Rd., Datong Dist., Taipei City
