# Supplementary material for: Patients as teachers: a qualitative study of spiritual care delivery experiences of senior healthcare providers in Taiwan
Source: BMC Med Educ. 2026 Feb 24;26:523. doi: 10.1186/s12909-026-08852-1 (PMC13037104; doi:10.1186/s12909-026-08852-1)
Supplement: Supplementary file 3 — Supplementary Material 3. [file 12909_2026_8852_MOESM3_ESM.pdf]

Table S2. Major themes and subthemes (summary of the Results)

Major Theme	Subtheme
1. Spiritual distress often manifests in subtle or culturally mediated ways	1.1 Uncontrolled symptoms with unknown cause<br>1.2 Disorganized expressions revealing inner chaos<br>1.3 Illness interpreted as punishment or burden<br>1.4 Culturally mediated expressions of spiritual distress
2. Building trust as a foundation for spiritual dialogue	2.1 Expressing needs in casual conversation<br>2.2 Presence and familiarity as essential elements
3. Facilitating meaning reconstruction through spiritual care delivery	3.1 Caregivers' responses shape the depth of the dialogue<br>3.2 Life review for integration and value reconstruction<br>3.3 Symbolic actions and rituals<br>3.4 Reconnection with others
4. Reflection and integration	4.1 Patients as teachers for professional and

into professional learning and personal growth	personal growth<br>4.2 Redefining professional roles and mission<br>4.3 Addressing challenges and educational needs
